# Predictors of morbidity in revisional bariatric surgery and bariatric emergencies at an MBSAQIP-accredited community hospital

**DOI:** 10.1186/s13017-022-00459-3

**Published:** 2022-10-29

**Authors:** Daniel Tomey, Alessandro Martinino, Joseph Nguyen-Lee, Alfred Lopez, Priya Shenwai, Zhuoxin Long, Jichong Chai, Tapan Nayak, James Wiseman, Rodolfo Oviedo

**Affiliations:** 1 Department of Surgery, Houston Methodist, 6550 Fannin St, SM1661, Houston, TX 77030 USA; 2 Faculty of Medicine, University of Zulia, Maracaibo, Venezuela; 3 Faculty of Medicine, Sapienza University of Rome, Rome, Italy; 4 Netaji Subhash Chandra Bose Medical College, Jabalpur, India; 5 Department of Statistics, George Washington University, Washington, DC, USA; 6 Department of Surgery, Johns Hopkins University, Baltimore, MD, USA; 7 Weill Cornell Medical College, Cornell University, New York City, NY, USA; 8 Texas A&M University College of Medicine, Bryan, TX, USA

**Keywords:** Revisional bariatric surgery, Bariatric emergencies, Community hospital, MBSAQIP

## Abstract

**Introduction:**

Bariatric surgery revisions and emergencies are associated with higher morbidity and mortality compared to primary bariatric surgery. No formal outcome benchmarks exist that distinguish MBSAQIP-accredited centers in the community from unaccredited institutions.

**Methods:**

A retrospective chart review was conducted on 53 bariatric surgery revisions and 61 bariatric surgical emergencies by a single surgeon at a high-volume community hospital accredited program from 2018 to 2020. Primary outcomes were complications or deaths occurring within 30-days of the index procedure. Secondary outcomes included operative time, leaks, surgical site occurrences (SSOs), and deep surgical site infections.

**Results:**

There were no significant differences in the demographic characteristics of the study groups. Mean operative time was significantly longer for revisions as compared to emergency operations (149.5 vs. 89.4 min). Emergencies had higher surgical site infection (5.7% vs. 21.3%, *p* < 0.05) and surgical site occurrence (SSO) (1.9% vs. 29.5%, *p* < 0.05) rates compared to revisions. Logistic regression analysis identified several factors to be predictive of increased risk of morbidity: pre-operative albumin < 3.5 g/dL (*p* < 0.05), recent bariatric procedure within the last 30 days (*p* < 0.05), prior revisional bariatric surgery (*p* < 0.05), prior duodenal switch (*p* < 0.05), and pre-operative COPD (*p* < 0.05).

**Conclusion:**

Bariatric surgery revisions and emergencies have similar morbidity and mortality, far exceeding those of the primary operation. Outcomes comparable to those reported by urban academic centers can be achieved in community hospital MBSAQIP-accredited centers.

## Introduction

Metabolic and bariatric surgery remains the most effective treatment for morbid obesity, and as such, the field has undergone rapid growth in recent years. Fewer than 5,000 bariatric procedures were performed worldwide in 1987; this number exceeded 350,000 in 2008 [1]. Along with the increase in these primary operations has come a commensurate growth in the number of related surgeries. In particular, the volume of revisional bariatric surgeries performed has doubled from 6% in 2011 to 13.6% in 2015 [2].

Revisional bariatric surgery (RBS) has evolved to address those patients whose original operation was unsuccessful in achieving satisfactory weight loss goals, or in whom complications from the original operation have occurred. Emergency bariatric surgery refers to those time-sensitive operations that arise because of the index operation, and include but are not limited to, internal hernias, intussusception, or perforated marginal ulcers. Moreover, both are associated with higher morbidity and mortality as compared to primary bariatric surgery. RBS has predominantly been characterized within the academic hospital environment, with the literature only recently exploring outcomes achieved in the community/rural hospital arena [3, 4].

Despite higher rates of complications, RBS is safe, particularly when performed in Metabolic and Bariatric Surgery Accreditation and Quality Improvement Program (MBSAQIP)-accredited centers. Mortality rates following revisional gastric sleeve or gastric bypass have been reported at less than 1%, although most of these data are derived from the experience of large academic centers [5, 6]. Less clear is the impact of a community hospital setting on outcomes associated with these procedures, although previous evidence has suggested they are comparable [7, 8]. On the other hand, very little is also known about the influence of a non-urban, community location on outcomes associated with emergency bariatric operations, even in accredited programs. Furthermore, there exists no consensus on the emergent management of bariatric surgery complications.

The objective of this study was to establish benchmarks for outcomes associated with RBS and emergency bariatric operations in the setting of an MBSAQIP-accredited community hospital and distinguish the worldwide importance of the general surgeon’s proficiency in bariatric emergency management regardless of metabolic and bariatric surgery fellowship training.

## Methods

A retrospective chart review was conducted to identify all RBS and emergent bariatric operations performed by a single, fellowship-trained metabolic and bariatric surgeon from 2018 to 2020. These were performed at a high-volume tertiary referral center in Winchester, Virginia, USA.

All procedures performed in a minimally invasive fashion utilized either a laparoscopic or robotic-assisted laparoscopic approach (da Vinci Xi or X platforms, Intuitive Surgical, Sunnyvale, CA, USA).

The primary outcomes measured were complications and deaths that occurred within the first 30-days after surgery. Secondary outcomes included operative time, leaks, and intra-abdominal abscess, and mean decrease in body mass index (BMI).

Software R, version 3 (R Foundation, Vienna, Austria) was used for the statistical analysis. For continuous variables, Student t-tests were used to detect significant differences between the two groups. Chi-squared tests were used for categorical variables with more than five observations, and Fisher’s exact test was utilized for variables with less than five observations. Logistic regression was conducted to identify patient characteristics associated with higher risk of morbidity. This study was conducted under the approval of the hospital’s Institutional Review Board, Protocol #20200104.

## Results

Table 1 shows the demographic characteristics of the study groups, including procedure type, bariatric surgical history, and comorbidities. The number of patients within the two groups was comparable (RBS = 53, emergent = 61). There was no significant difference in the mean age (RBS = 49, emergent = 50), mean preoperative albumin (RBS = 3.59, emergent = 3.41), duration of follow up duration (RBS = 5 months, emergent = 6 months) and American Society of Anesthesiology classification (RBS = 2.88, emergent = 2.93). The most common surgical approach for the revisional cases was laparoscopic like in the emergent surgery group. A total of 8 out of 53 revisional operations were performed utilizing the robotic platform, compared to none of the emergent cases. The most common index bariatric procedure among the revisional group was Roux-en-Y Gastric Bypass (RYGB, including RYGB + band gastric pouch) followed by sleeve gastrectomy, and adjustable gastric band. Similarly, the most common index procedure among the emergent patients were prior RYGB, followed by duodenal switch.

**Table 1:** Patient demographics

Covariates	Revision (*N* = 53)	Emergency (*N* = 61)	*p* value
Age	49.15 (11.59)	49.64 (12.48)	0.8313
Pre-op albumin (gm/dL)	3.59 (0.63)	3.41 (0.80)	0.2121
Pre-op BMI (kg/m^2^)	36.78 (10.25)	34.49 (10.35)	0.243
Follow up duration	5.15 (3.65)	6.23 (5.02)	0.1925
ASA (median)	2.88 (0.59)	2.93 (0.63)	0.5633
Sex			
Female	46	52	0.8126
Male	7	9	
Procedure type*			
Laparoscopic	44	56	** < 0.05**
Robotic	8	0	
Open	1	5	

Bariatric surgery history	N	%	N	%	
Recent bariatric procedure within last 30 days*	2	3.77	17	27.87	** < 0.05**
Prior RYGB* (+Prior RYGB with band gastric pouch)	25	45.28	53	86.89	** < 0.05**
Prior SG*	10	18.87	1	1.64	** < 0.05**
Prior DS	4	7.55	7	11.48	0.4786
Prior AGB*	9	16.98	2	3.28	** < 0.05**
Prior VBG	4	7.55	1	1.64	0.1817
Prior revisional bariatric surgery	7	13.21	12	19.67	0.3556
Comorbidities					
Pre-op DM2	10	18.87	12	19.67	0.9136
Pre-op HTN	18	33.96	15	24.59	0.2711
Pre-op OSA	14	26.42	23	37.70	0.1991
Pre-op COPD	1	1.89	2	3.28	1
Pre-op dyslipidemia	17	32.08	17	27.87	0.6244
Pre-op GERD	39	73.58	37	60.66	0.1441
Pre-op CAD	6	11.32	5	8.20	0.5731
Pre-op CHF	1	1.89	1	1.64	1
Pre-op CKD/ESRD	5	9.43	1	1.64	0.09507
History of Tobacco use	22	41.51	24	39.34	0.8142
Pre-op dyslipidemia	3	5.66	1	1.64	0.3362
Pre-op GERD	1	1.89	1	1.64	1

The values in bold characters correspond to statisfically significant differences with a p value < 0.05 according to biostatistical standards

Table 2 shows a comparison of the mortalities of the two groups. There was no statistically significant difference between the two groups at 30 days or overall.

**Table 2:** Primary outcomes

Primary outcomes	Revision (*N* = 53)	Emergency (*N* = 61)	*P* value
0-day morbidity	19 (35.85)	29 (47.54)	0.2073
Mortality	2 (3.77)	1 (1.64)	0.5967

Table 3 demonstrates the observed secondary outcomes within the two groups. Mean operative times were substantially lower in the emergency surgeries (149.5 min vs. 89.4 min. A greater decrease in BMI was observed within the RBS group as compared to the emergent group (-4.86 vs. -2.63, *p* < 0.05). SSIs were seven times more common in the emergent group (21% vs. 3%, *p* < 0.05). Similarly, there was a significantly greater incidence of postoperative seroma in the emergency group compared.

**Table 3:** Secondary outcomes

Secondary outcomes:	Revision (*N* = 53)	Emergency (*N* = 61)	*p* value
Intraoperative time (min)*	149.51 (66.80)	89.44 (45.24)	** < 0.05**
EBL	83.21 (199.43)	35.57 (36.46)	0.09517
Length of stay (days)	4.84 (7.39)	6.52 (8.29)	0.2616
Post-op BMI (kg/m^2^)	31.91 (7.96)	31.86 (7.65)	0.9705
Change in BMI (BMI points)*	− 4.86(5.78)	− 2.63 (6.07)	** < 0.05**

	*N*	%	*N*	%	
Conversion to open	1	1.89	6	9.84	0.1199
Post op superficial SSI*	3	5.66	13	21.31	** < 0.05**
Post op intra-abdominal hemorrhage/staple line bleeding	3	5.66	2	3.28	0.6622
Post op GI bleeding	1	1.89	1	1.64	1
Post op abdominal wall seroma *	1	1.89	18	29.51	** < 0.05**
Post op abdominal wall hematoma	2	3.77	5	8.20	0.4468
Unexpected return to OR within 30 days	8	15.09	6	9.84	0.3936
Expected (staged) return to OR within 30 days	2	3.77	4	6.56	0.6839
Post-op SBO	3	5.66	5	8.20	0.7223
Post-op hollow viscus perforation	4	7.55	1	1.64	0.1817
Post-op endoscopy	16	30.19	13	21.31	0.2777
Anastomotic or staple line leak	1	1.89	1	1.64	1
Stricture requiring OR	2	3.77	1	1.64	1
Post op marginal ulcers	14	26.42	8	13.11	0.07269
Stricture requiring endoscopic balloon dilation *	8	15.09	0	0.00	0.00165
Post-op intra-abdominal abscess	9	16.98	10	16.39	0.9331
Readmission within 30 days	10	18.87	10	16.39	0.729
ED visits within 30 days	15	28.30	17	27.87	0.9591
Infusion center visit for dehydration	5	9.43	4	6.56	0.7312
Post-op DVT/PE	1	1.89	2	3.28	1
C diff colitis	5	9.43	4	6.56	0.7312
Toxic megacolon	1	1.89	1	1.64	1
Post-op MI	2	3.77	1	1.64	0.5967
Post-op pneumonia	3	5.66	7	11.48	0.3342
Post-op AKI	6	11.32	14	22.95	0.1034
Subsequent surgery > 30 days	11	20.75	14	22.95	0.7774

The values in bold characters correspond to statisfically significant differences with a p value < 0.05 according to biostatistical standards

Table 4 shows the indications for surgery and the type of surgery performed. The most common indication for intervention in the RBS group was ulcer disease refractory to medical management (*N* = 11, 20.8%), with the common surgery performed being laparoscopic gastrojejunostomy (GJ revision) (*N* = 11, 20.8%). By contrast, the most frequent indication for surgery in the emergent group was small bowel obstruction due to internal hernia (*N* = 28, 45.9%), with the most common operation being closure of internal hernia (*N* = 34, 54.0%).

**Table 4:** Most common indications and operations

Categories	Bariatric revisions (*n* = 53)	Bariatric emergencies (*n* = 61)
Most frequent indications	GI Ulcer refractory to medical management *n* = 11 (20.76%)	SBO from internal hernia *n* = 28 (45.90%)
	GI ulcer w/obstruction *n* = 5 (9.43%)	SBO from adhesions * n* = 25 (40.98%)
	Gastric Sleeve with severe GERD *n* = 5 (9.43%)	Hollow viscus perforation (after recent primary procedure) *n* = 5 (8.20%)
Most frequent operations	Laparoscopic GJ revision *n* = 13 (24.53%)	Lysis of adhesions *n* = 44 (72.13%)
	Laparoscopic SG to RYGB *n* = 7 (13.21%)	Internal hernia closure *n* = 34 (55.73%)
	Laparoscopic ABG removal *n* = 4 (7.55%)	Exploration for suspicion for complications after recent primary procedure *n* = 12 (19.67%)

The results of the logistic regression analysis are included in Table 5. Independent predictors of any morbidity or mortality in the first 30 postoperative days following RBS or emergent bariatric surgery included: preoperative albumin level less than 3.5 g/dL (*p* < 0.05), history of index procedure within the previous 30 days (*p* < 0.05), prior history of RBS (*p* < 0.05), prior history of duodenal switch (DS) (*p* < 0.05), and history of chronic obstructive pulmonary disease (*p* < 0.05).

**Table 5:** Logistic regression analysis for combined bariatric revisions and bariatric emergencies

Covariate	Estimate	*P* value
Revision	0.205	0.67138
Pre-op albumin	− 1.489	0.00035***
Recent bariatric procedure within last 30 days	1.984	0.01052*
Prior revisional bariatric surgery	1.716	0.02212*
Prior DS	− 2.541	0.01121*
Pre-op COPD	17.686	0.04554*
Open	16.607	0.07058

Significance codes: ‘***’ 0.001, ‘**’ 0.01, ‘*’ 0.05, ‘.’ 0.1

The values in bold characters correspond to statisfically significant differences with a p value < 0.05 according to biostatistical standards

## Discussion

Revisional bariatric surgery is a technically demanding super-specialty of metabolic and bariatric surgery associated with higher complication rates and mortality as compared to primary bariatric surgery. The surgical treatment of bariatric emergencies requires a similarly demanding and specialized skill set but has yet to receive wide recognition as a distinct component of metabolic and bariatric surgery [7, 9, 10]. We understand these two disciplines to be uniquely interrelated, with common themes and overlapping knowledge bases. Understanding predictors of undesirable outcomes within each of these realms is a critical determinant of competence for the dedicated metabolic and bariatric surgeon. Moreover, as the prevalence of bariatric operations continues to surge, so too will the demand for centers with demonstrated proficiency in both RBS and the management of emergencies unique to the field. This study is the first to our knowledge to establish outcome benchmarks for RBS and emergent bariatric surgery within a community hospital MBSAQIP-accredited center.

Although the MBSAQIP is an accreditation and quality improvement system that is relevant in North America, its influence is evident in other parts of the world, since the American Society for Metabolic and Bariatric Surgery (ASMBS) is a member organization of the International Federation for the Surgery of Obesity and Metabolic Disorders (IFSO), which is a global society. Therefore, the outcomes and quality data derived from accredited programs can be emulated by programs around the world that are looking for standardization and systematic quality improvement processes. On the other hand, bariatric surgical emergencies are common, and with the increased performance of bariatric procedures, general surgeons who may not be familiar with the technical details of metabolic surgery may be called upon to operate on patients in an emergency situation for whom life-saving measures must be taken. It becomes crucial, as a result, to be familiar with these concepts and to understand that bariatric emergencies or semi-urgent revisions have a high morbidity and mortality index compared to primary surgery. Finally, bariatric revisions are oftentimes the cause of potential emergencies such as those from perforated ulcers due to gastrojejunostomy ulcers, or internal hernias from a Roux en Y gastric bypass revision or a second-stage duodenal switch.

Accreditation of MBSAQIP-accredited centers is the function of a joint effort between the American College of Surgeons and the American Society for Metabolic and Bariatric Surgery [11]. To achieve accreditation, institutions must meet specific standards with respect to training, outcome measurement/reporting, and resource allocation. Conspicuously absent from these standards are metrics specific to the patient rescue when the index operation either fails or a complication arises. Little is known regarding the impact of accreditation on outcomes in either RBS or emergent bariatric surgery. In 2018, Qiu and colleagues reported an overall complication rate of 14.3% for patients undergoing RBS in an urban accredited center [12]. In a 2014 review by Brethauer and coauthors reported complication rates related to RBS ranging from 14.6 to 33.0%. This correlates well with the complication rates observed in our study [13].

Even less is known about the impact of accreditation on outcomes in the surgical treatment of bariatric emergencies. A 2018 study out of France evaluated the impact of centralized management of bariatric surgery complications on 90-day mortality. The authors noted a statistically significant improvement in mortality associated with centralization and were further able to attribute this to improved management rather than a lower observed incidence of complications [14]. This observation reinforces the idea that outcomes associated with the management of bariatric emergencies benefit from specialized institutions. Moreover, our data suggest that treatment outcomes are comparable even when provided in rural settings, suggesting that accreditation, not location, may be the key driver of these results.

The emergency setting should not be an absolute contraindication to performing robotic surgery. The robotic approach in the emergency context is considered safe, feasible, and is associated with positive safety outcomes that are derived from the minimally invasive surgical approach. However, it is true that availability and accessibility of the robotic platform for the emergency setting is limited, especially on night shifts [15]. In addition, during day shifts it may also become difficult to obtain access to the robotic platform for emergencies due to the high likelihood that it is being used by other surgeons for elective procedures. It becomes crucial, as a result, for the surgeons to be proficient at performing these operations with the conventional laparoscopic approach in case the robotic technology is not available, or the conditions are not ideal for its implementation.

There is a growing need for non-fellowship trained bariatric surgeons to be familiar with bariatric emergencies which are being addressed by more general surgeons seeking training in Minimally Invasive Surgery (MIS). Ideally, bariatric emergency and revisional surgery will be best handled by surgeons with fellowship training or those who did not go to fellowship but have dedicated themselves to the practice of metabolic and bariatric surgery over the years as their focused practice designation (FPD). Since bariatric procedures are growing in numbers, there is a need for improved surgical management of bariatric emergencies and revisional surgeries in the general surgeon's field.

The mortality rate for both RBS and emergent bariatric surgery in this study was miniscule, congruent with existing literature. Accreditation has long been known to be an independent predictor of mortality in bariatric surgery, and this observation is not particularly noteworthy, given that all these procedures took place in an accredited institution [16]. Interestingly, the most important predictor of postoperative complication in the RBS group in the present study was preoperative GERD. This factor had no predictive value at all in the emergent patients. The reason for this is unclear but may have implications regarding the technical quality of the original operation.

Mean operative time for RBS in this study was substantially longer than in emergent operations. This observation may be due to the technical challenges posed by revisional metabolic surgery including the presence of dense adhesions in the preoperative field in addition to the careful evaluation of anastomosis creation or endoscopic leak test performance. In comparison, emergency bariatric procedures did not commonly include these challenges.

This observation has implications in terms of the concentration of management of these complications within accredited centers. Moreover, the rate of intraabdominal abscess between the two types of procedures was equivalent. This observation has not been previously reported, and there is no clear explanation for it. Future directions might compare culture results to better understand the significance of this observation.

The rates of SSI and SSO were significantly higher in the emergent group. In the setting of emergency bariatric surgeries, the patients were less optimized in terms of nutrition and pre-operative measures. In the context of a surgical emergency, inadequate glycemic control, the more significant inflammatory response, and factors associated with a non-elective, planned operation play a role in the higher index of SSI and SSO. Both occurrences impose a burden in terms of cost of care, as well as the risk of abdominal wall hernia. Recognizing this risk and taking steps to mitigate them is an important component of establishing the expertise needed to bolster accreditation.

Previous studies have shown that the robotic and laparoscopic platforms yield similar outcomes in RBS [17, 18]. Conversely, little is known about the roles of various approaches in the management of bariatric emergencies. Our study is among the first to demonstrate the two and to evaluate outcomes in a community hospital MBSAQIP-accredited program.

The most important covariates that were associated with higher morbidity and mortality in both groups are preoperative albumin, recent bariatric procedures within the last 30 days, prior revisional bariatric surgery, and pre-op COPD. These variables have also been identified in other literature. Procedure type is also significant since a robotic approach predicts a lower risk than a laparoscopic approach in our revision data. In contrast, an open approach predicts a higher risk than a laparoscopic approach in our emergency data and combined data [20].

On regression analysis, no significant difference was noted in rates of morbidity and mortality between the two groups. There were, however, some interesting caveats. Prior RYGB proportion differed significantly between the two groups. We would have expected this observation to contribute to a difference in complication rates, but this was not the case. This could be due to the study being underpowered to detect a significant effect here. We propose that this may be explained by the following two covariates: recent bariatric procedure within the last 30 days and procedure type. Recent bariatric procedure within 30 days is one of the most important covariates associated with the primary outcome. However, the number of patients is quite different between the two groups (2 vs. 17), with a p-value 0.0007 (Table 1). This may affect the morbidity and mortality between two groups. Procedure type also plays a role. In the revision group, most of the procedures were laparoscopic and robotic, while in the emergency group, all the procedures were laparoscopic and open. However, robotics is negatively associated with primary outcome, while open is positively associated with it. In laparoscopic surgery, the 30-day morbidity between two groups is very close (40.91% vs. 42.86%). Therefore, the morbidity may be influenced by the big difference between the number of robotic (8 vs. 0) and open (1 vs. 5) surgeries between the two groups.

*World Journal of Emergency Surgery* was selected as the appropriate journal to share the results from this study due to its impact around the world. WJES exerts a positive influence on the general surgery community that treats patients with bariatric surgical emergencies and semi-urgent revisions with their corresponding high morbidity and mortality. In addition, bariatric emergencies are common and relevant not just for sub-specialized bariatric surgeons, but also for general surgeons. As a result, there is value in reporting our experience on these emergent procedures to learn from our observations and study a topic that has not been explored in detail in the literature**.**

Future directions for research that may arise from the lessons learned through this experience may include an investigation into any significant predictors of postoperative emergency department visits after bariatric surgery. The ability to determine which patients are at increased risk of repeated visits to the emergency department could help ease this healthcare cost burden by allowing more frequent and targeted interventions on postoperative follow-up [19–21].

It would be an interesting research subject for further investigations on revisional and emergency topics, and the outcomes for emergency bariatric patients who are taken care of by non-bariatric emergency surgeons. Moreover, it would be important to review the current literature on the non-bariatric surgery trained general surgeon's outcomes and the results in emergency bariatric surgeries. This topic is relevant due to the insufficient availability of surgeons (bariatric and general) in rural community hospitals.

## Limitations

This study has several limitations. First, as with many single-institution studies within the surgical literature, group sizes are relatively small. This research is ongoing, and relationships not found to be statistically significant in the present effort may ultimately prove to demonstrate effects not detected in this retrospective study. This issue poses a limitation in terms of generalizing the applicability of the outcomes to the community and urban academic centers. Multi-institutional prospective studies represent the ideal form of research and would be useful for corroborating the observations in this single-institution retrospective study. Another limitation of this study lies in its design. The inclusion of a single institution imposes constraints with respect to the generalizability of these observations. It is worth noting, however, that having successfully navigated the accreditation process implies a uniformity of standards that support the notion of generalizability here. Similarly, another limitation is that all the operations were performed by a single fellowship-trained minimally invasive metabolic and bariatric surgeon, which also has implications in terms of generalizability.

## Conclusion

From our single institutional study, bariatric surgery revisions and emergencies have similar morbidity and mortality in a high-volume community hospital MBSAQIP-accredited center. Revisions have a longer intraoperative time and higher anastomotic stricture incidence requiring endoscopic dilation, but a lower incidence of SSI and SSO compared to emergencies. Factors associated with higher morbidity include prior revisional surgery, prior DS, pre-op COPD, abnormally low pre-op albumin, and recent bariatric procedures within the last 30 days.

## Data Availability

The de-identified data and tables corresponding to the retrospective chart review are available upon request and kept in a secure location.

## Abbreviations

RYGB: Roux-en-Y gastric bypass
SG: Sleeve gastrectomy
DS: Duodenal switch
ABG: Adjustable gastric band
VBG: Vertical banded gastroplasty
GJ: Gastrojejunostomy
BMI: Body mass index
DM2: Type 2 diabetes mellitus
HTN: Hypertension
OSA: Obstructive sleep apnea
COPD: Chronic obstructive pulmonary disease
GERD: Gastroesophageal reflux disease
CAD: Coronary artery disease
CHF: Congestive heart failure
CKD: Chronic kidney disease
ESRD: End stage renal disease
ASA: American Society of Anesthesiologists
OR: Operating room
SSI: Surgical site infection
SSO: Surgical site occurrence
EBL: Estimated blood loss
GI: Gastrointestinal
SBO: Small bowel obstruction
DVT: Deep venous thrombosis
PE: Pulmonary embolus
MI: Myocardial infarction
C diff: Clostridium difficile
